# Spinal TRPA1 Contributes to the Mechanical Hypersensitivity Effect Induced by Netrin-1

**DOI:** 10.3390/ijms23126629

**Published:** 2022-06-14

**Authors:** Hong Wei, Liisa Ailanen, Miguel Morales, Ari Koivisto, Antti Pertovaara

**Affiliations:** 1 Department of Physiology, Faculty of Medicine, University of Helsinki, 00014 Helsinki, Finland; hong.wei@helsinki.fi; 2 Research and Development, Orion Corporation Orion Pharma, 20101 Turku, Finland; liisa.ailanen@orionpharma.com (L.A.); miguel.morales@orionpharma.com (M.M.); ari-pekka.koivisto@orionpharma.com (A.K.)

**Keywords:** netrin-1, TRPA1 receptors, mechanical hypersensitivity, medullary pain control neuron, spinal cord

## Abstract

Netrin-1, a chemoattractant expressed by floor plate cells, and one of its receptors (deleted in colorectal cancer) has been associated with pronociceptive actions in a number of pain conditions. Here, we addressed the question of whether spinal TRPC4/C5 or TRPA1 are among the downstream receptors contributing to pronociceptive actions induced by netrin-1. The experiments were performed on rats using a chronic intrathecal catheter for administration of netrin-1 and antagonists of TRPC4/C5 or TRPA1. Pain sensitivity was assessed behaviorally by using mechanical and heat stimuli. Effect on the discharge rate of rostral ventromedial medullary (RVM) pain control neurons was studied in lightly anesthetized animals. Netrin-1, in a dose-related fashion, induced mechanical hypersensitivity that lasted up to three weeks. Netrin-1 had no effect on heat nociception. Mechanical hypersensitivity induced by netrin-1 was attenuated by TRPA1 antagonist Chembridge-5861528 and by the control analgesic compound pregabalin both during the early (first two days) and late (third week) phase of hypersensitivity. TRPC4/C5 antagonist ML-204 had a weak antihypersensitivity effect that was only in the early phase, whereas TRPC4/C5 antagonist HC-070 had no effect on hypersensitivity induced by netrin-1. The discharge rate in pronociceptive ON-like RVM neurons was increased by netrin-1 during the late but not acute phase, whereas netrin-1 had no effect on the discharge rate of antinociceptive RVM OFF-like neurons. The results suggest that spinal TRPA1 receptors and pronociceptive RVM ON-like neurons are involved in the maintenance of submodality-selective pronociceptive actions induced by netrin-1 in the spinal cord.

## 1. Introduction

Netrin-1 is a chemoattractant expressed by floor plate cells that can both promote and orient commissural axon outgrowth [1]. Deleted in colorectal cancer (DCC) and neogenin are receptors mediating attractive signaling by netrin-1 [2,3], whereas Unc5 receptors are mediating repulsive signaling by netrin-1 [4]. There is accumulating evidence that netrin-1 and DCC exert a pronociceptive role in various pain conditions. Patients with chronic low back pain were shown to have increased expression of netrin-1 and DCC immunopositive cells in their lumbar intervertebral discs, suggesting that netrin-1 might have a pronociceptive role in the low back area [5]. A chemically-induced experimental animal model of neuropathic pain induced upregulations of netrin-1 and DCC in the spinal dorsal horn associated with primary afferent sprouting and mechanical allodynia, indicating that netrin-1 and DCC play a role in neuropathic pain [6]. In line with this, chronic constriction injury in mice induced neuropathic pain behavior that was associated with increased levels of spinal netrin-1 and DCC, whereas spinal administration of netrin-1 in control animals induced upregulation of DCC and pain hypersensitivity [7]. Experimental animal studies indicate that netrin-1 may also play a role in postoperative [8] and arthritic pain [9,10]. Studies on samples of osteoarthritis patients suggest that osteoclasts secrete excessive amounts of netrin-1 and induce sensory innervation and pain in the arthritic joint [10]. In addition, human studies have reported that in patients with endometriosis, netrin-1 and DCC levels are increased in endometrial tissues [11], and patients with small fiber neuropathy have increased netrin-1 gene expression in keratinocytes [12]. In genome-wide analysis, the DCC netrin-1 receptor gene was identified as the top gene associated with multisite pain [13].

Little is known about potential receptors other than DCC that might exert a role in mediating the pronociceptive effects of netrin-1. Here we tested the hypothesis that among downstream mechanisms mediating pronociceptive effects of netrin-1 are transient receptor potential ankyrin 1 (TRPA1) [14,15], and/or transient receptor channel 4/5 (TRPC4/C5) [16,17], that have been shown to mediate pronociceptive actions in various pain conditions (see for reviews [18,19]). Our hypothesis was based on the following findings. Earlier studies indicate that netrin-1 has multiple actions, among which is a dichotomous role in inflammation [20]. A proinflammatory effect of netrin-1 [21,22,23,24] might generate various endogenous agonists of TRPA1 [18] and TRPC4/C5 [19]. Moreover, netrin receptors DCC, TRPC4/C5, and TRPA1 mRNA are colocalized in a number of human and rodent sensory neuron subtypes [25,26]. Further, it is possible that macrophages play a role in DCC-induced pain phenotype as macrophage-derived netrin was recently shown to contribute to endometriosis-associated pain [11]. To test our working hypothesis, we determined whether pain behavior induced by spinal administration of netrin-1 could be reversed with antagonists of TRPA1 or TRPC4/C5. Since studies assessing the chronification of pain have shown that mechanisms underlying the early phase of pain may differ from those underlying the late phase of pain [27], the experiments were performed both during the early and late phases following netrin-1 treatment. In a parallel electrophysiological study, we assessed whether spinal administration of netrin-1 is accompanied by early or late changes in the discharge properties of pain control neurons of the rostral ventromedial medulla (RVM).

## 2. Results

### 2.1. Test Stimulus-Evoked Pain Behavior following Spinal Administration of Netrin-1

**Figure 1:**
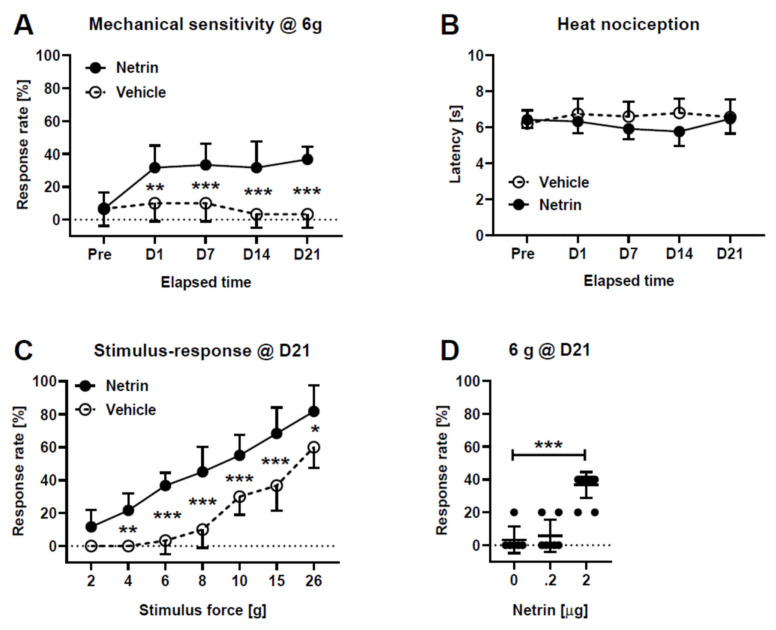
Mechanical sensitivity (**A**,**C**,**D**) and heat nociception (**B**) after intrathecal administration of netrin-1. (**A**) shows limb withdrawal response tested at the force of 6 g at various time points. (**B**) shows paw flick latency to heat at various time points. (**C**) shows limb withdrawal response to various test stimulus forces 21 days after treatment. (**D**) shows the effect of drug dose on the behavioral effect 21 days after treatment. The dose of netrin-1 in (**A**–**C**) was 2.0 µg. D1–D21 indicate days after treatment. The graphs show mean values and the error bars represent S.D. (n_Net2.0_ = 12, n_Net0.2_ = 7, n_Veh_ = 6). In the *Y*-axis, increase in the response rate to mechanical stimulation or decrease in the latency to heat stimulation represent hypersensitivity effect. In (**A**,**C**), n_Netrin_ = 12, n_Vehicle_ = 6. In (**B**), n_Netrin_ = 7 and n_Vehicle_ = 6. In (**D**), n_0_ = 6, n_.2_ = 7, n_2_ = 12. * *p* < 0.05, ** *p* < 0.01, *** *p* < 0.005 (*t*-test with Bonferroni correction; reference: unless specified, the corresponding value in the Vehicle group).

A single injection of netrin-1 (2.0 µg) induced a long-lasting mechanical hypersensitivity effect that lasted up to three weeks (main effect of netrin-1: *F*_1,16_ = 30.7, *p* < 0.0001; Figure 1A) and varied with elapsed time (interaction between time and netrin-1 treatment: *F*_4,64_ = 7.0, *p* < 0.0001). *Post hoc* tests indicated that mechanical hypersensitivity was significant from the first treatment day until day 21 (Figure 1A). On day 21, the traditional withdrawal threshold determined using psychometric function curves was 20.8 g ± 4.5 g in the control group and 10.5 g ± 4.4 g in the group treated with netrin-1 (*t*_14_ = 4.5, *p* = 0.0005). The pronociceptive effect of netrin-1 was submodality-dependent, since spinal administration of netrin-1 (2.0 µg) did not influence heat nociception (main effect of netrin-1: *F*_4,55_ = 0.4; Figure 1B). The mechanical hypersensitivity effect induced by 2.0 µg of netrin-1 (main effect of netrin-1: *F*_6,77_ = 53.1, *p* < 0.0001) did not vary significantly, independent of whether the test stimulus was applied at a low (≤6 g) or high intensity (interaction between test stimulus force and netrin-1 treatment: *F*_6,35_ = 2.3, *p* = 0.052; Figure 1C). The mechanical hypersensitivity effect induced by spinal administration of netrin-1 was dose-related (main effect of spinal treatments: *F*_2,22_ = 45.0, *p* < 0.0001; Figure 1D). *Post hoc* testing indicated that spinally administered netrin-1-induced a mechanical hypersensitivity effect at the dose of 2.0 µg but not at the dose of 0.2 µg (Figure 1D).

### 2.2. Attempts to Reverse Early and Late Mechanical Hypersensitivity Induced by Netrin-1

**Figure 2:**
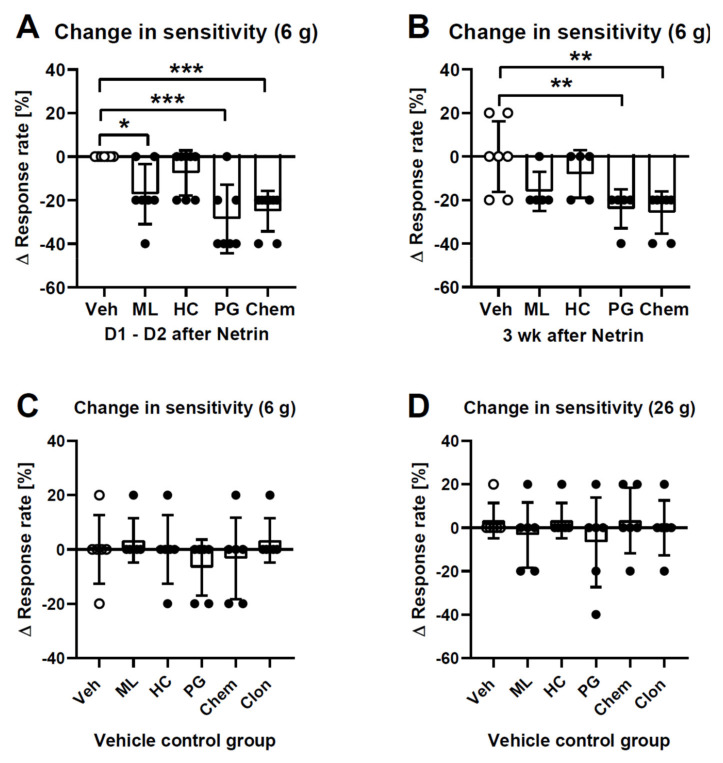
Attempts to reduce mechanical hypersensitivity induced by intrathecal administration of 2.0 µg of netrin-1 with various compounds during the first two days (**A**) and during the third week (**B**) of hypersensitivity. Effect of the studied intrathecally-administered compounds on mechanical sensitivity in healthy control animals assessed at a low (6 g; (**C**)) and high (26 g; (**D**)) test stimulus intensity. Veh = vehicle, ML = ML-204 (10 µg), HC = HC-070 (19 ng), PG = pregabalin (30 µg), Chem = Chembridge-5861528 (10 µg), Clon = clonidine (10 µg). Each bar represents the mean change in the withdrawal response to repeated application of the test stimulus at the force of 6 g (**A**–**C**) or 26 g (**D**) 15 min following intrathecal administration of the studied compound; 0% represents the response rate before administration of the studied compound, and response rate changes < 0% represent an antihypersensitivity effect induced by the tested compound. In (**A**), n = 7, except n_HC/Chem_ = 8. In (**B**), n_Veh/Chem_ = 7 and in other groups n = 5. In (**C**,**D**), n = 6 in all groups. The error bars represent S.D. * *p* < 0.05, ** *p* < 0.01, *** *p* < 0.005 (*t*-test with Bonferroni correction). The absolute response rates in the vehicle groups to stimulation at a force of 6 g were 40% ± 20% in (**A**), 31% ± 10% in (**B**), 13% ± 10% in (**C**), and to stimulation at a force of 26 g was 80% ± 13% in (**D**).

During the early phase (24–48 h after spinal treatment with 2.0 µg of netrin-1), spinal co-administration of the studied compounds had a significant main effect on mechanical hypersensitivity induced by netrin-1 (*F*_4,32_ = 8.3, *p* = 0.0001; Figure 2A). *Post hoc* testing indicated that mechanical hypersensitivity induced by netrin-1 in the early phase was significantly attenuated by ML-204 (10 µg; TRPC4/C5 antagonist), pregabalin (30 µg; an established analgesic control compound), and Chembridge-5861528 (10 µg; TRPA1 antagonist), but not by HC-070 (19 ng; TRPC4/C5 antagonist).

During the late phase (three weeks after spinal treatment with 2.0 µg of netrin-1), spinal co-administration of the studied compounds had a significant effect on mechanical hypersensitivity induced by netrin-1 (*F*_4,24_ = 6.4, *p* = 0.0011; Figure 2B). *Post hoc* testing indicated that in the late phase, mechanical hypersensitivity induced by netrin-1 was significantly attenuated by pregabalin (30 µg) and Chembridge-5861528 (10 µg), but not by ML-204 (10 µg) or HC-070 (19 ng) (Figure 2B). 

The drugs that were used in attempts to attenuate mechanical pain hypersensitivity induced by netrin-1 were also tested at the same dose in control animals. The studied drugs (ML-204, HC-070, pregabalin, Chembridge-5861528, or clonidine) had no significant effect on mechanical sensitivity in control animals, independent of whether the test stimulus force was low (6 g; main effect of studied drugs: *F*_5,30_ = 0.7; Figure 2C) or high (26 g; main effect of studied drugs: *F*_5,30_ = 0.5; Figure 2D).

### 2.3. Assessment of Ongoing Pain-Like and Anxiety-Like Behavior during Late Phase

**Figure 3:**
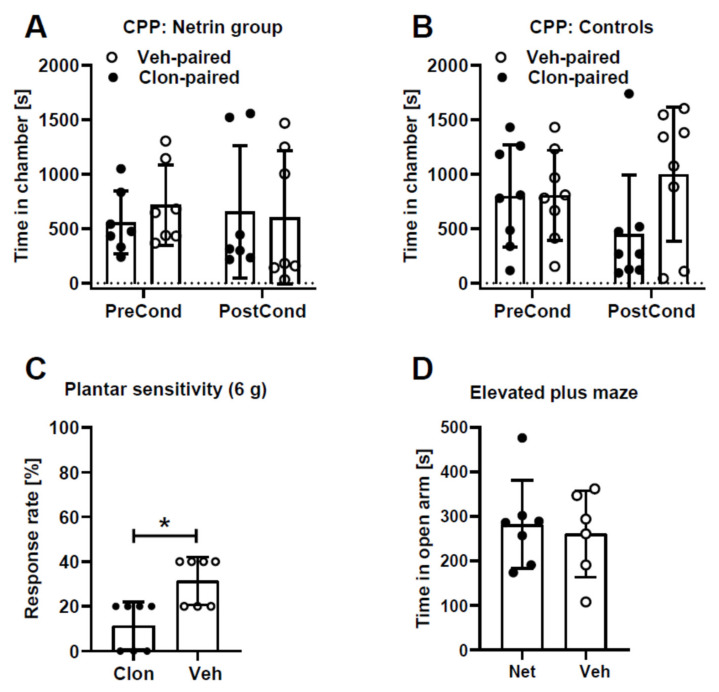
Clonidine-induced effect on conditioned place preference (CPP) in animals treated with netrin-1 (**A**) or vehicle (**B**), and on mechanical hypersensitivity following netrin-1 treatment (**C**). The time spent in the open arm of the elevated plus maze in animals treated with netrin-1 or vehicle (**D**). In (**A**,**C**), all animals were three weeks earlier treated intrathecally with 2.0 µg of netrin-1. Clon = clonidine (10 µg), Veh = vehicle (in (**C**), administered 15 min earlier; in (**D**), administered three weeks earlier), PreCond = before conditioning, PostCond = after conditioning, Net = netrin-1 (2.0 µg administered three weeks earlier). In (**A**,C,D), n = 7. In (**B**), n = 8. Error bars represent S.D. * *p* < 0.05 (paired *t*-test).

The conditioned place preference paradigm was used to assess whether animals have ongoing pain during the late phase (three weeks) of mechanical hypersensitivity induced by netrin-1 treatment. One of the test chambers was paired with a spinal administration of clonidine (α_2_-adrenoceptor agonist) at a dose (10 µg) that induced a significant antihypersensitivity effect in animals treated with netrin-1 (Figure 3C) but had no effect on mechanically-evoked responses in animals treated with vehicle (Figure 2C,D). It was expected that if the attenuation of hypersensitivity is associated with the attenuation of ongoing pain, the animals would prefer the clonidine-paired chamber after conditioning. However, this did not happen (main effect of clonidine: *F*_1,6_ = 0.1; interaction between clonidine treatment and conditioning: *F*_1,6_ = 0.2; Figure 3A), nor did clonidine induce place preference in vehicle-treated control animals (main effect of clonidine: *F*_1,7_ = 1.7; interaction between clonidine treatment and conditioning: *F*_1,7_ = 1.8; Figure 3B). 

An elevated plus maze test was used to assess whether netrin-1 treatment induces anxiety-like behavior during the late phase of hypersensitivity (three weeks). Time spent in the open arm of the elevated plus maze, however, was not different between animals treated with netrin-1 and vehicle (Figure 3D).

### 2.4. Comparison of Pain Behavior with Discharge Rates of Medullary Pain Control Neurons

#### 2.4.1. Acute Effect 30 Min after Administration of Netrin-1

**Figure 4:**
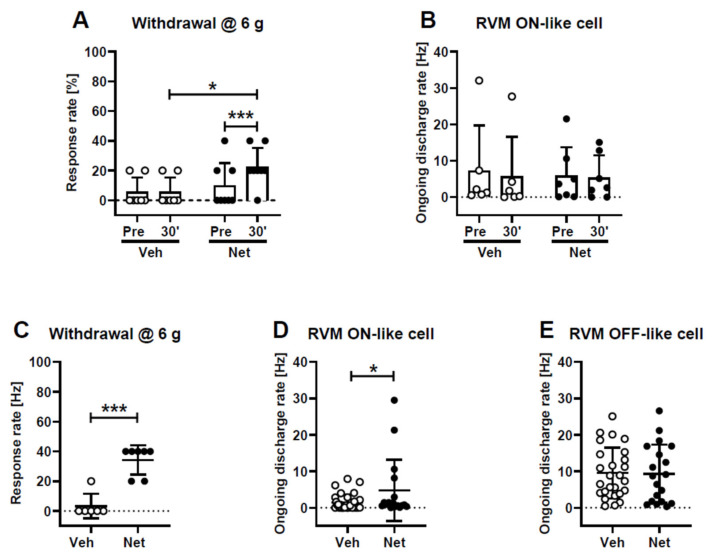
Acute (**A**,**B**) and chronic (**C**–**E**) effects of intrathecally-administered netrin-1 (Net, 2.0 µg) or vehicle (Veh) on mechanical sensitivity (**A**,**C**) and the response rates of presumably pronociceptive ON-like and antinociceptive OFF-like neurons in the rostral ventromedial medulla (RVM; **B**,**D**,**E**). In the upper graphs, effect of netrin-1 was studied before (Pre) and 30 min after its administration, whereas the lower graphs represent results obtained three weeks after treatment. The graphs show mean results and the error bars represent S.D. * *p* < 0.05, *** *p* < 0.005 (in (**A**), *t*-test with a Bonferroni correction; in (**C**), unpaired *t*-test).

The behavioral assessment of mechanical sensitivity before and 30 min after the spinal administration of netrin-1 (2.0 µg) or vehicle in awake animals indicated that the treatment induced a significant main effect (*F*_1,13_ = 10.1, *p* = 0.007) that varied with the treatment group (interaction: *F*_1,13_ = 10.1, *p* = 0.007; Figure 4A). *Post hoc* testing indicated the development of a significant mechanical hypersensitivity effect within 30 min in the group treated with netrin-1, but not in the vehicle control group (Figure 4A).

The electrophysiological in vivo recording of pronociceptive rostral ventromedial medullary (RVM) ON-like pain control neurons (n_Netrin_ = 7, n_Vehicle_ = 6) in lightly anesthetized animals (n_Netrin_ = 6, n_Vehicle_ = 5) indicated that the ongoing discharge rate was not significantly changed 30 min after treatment with netrin-1 or vehicle (main effect of elapsed time: *F*_1,11_ = 2.9), independent of the treatment group (interaction: *F*_1,11_ = 0.7; Figure 4B).

#### 2.4.2. Chronic Effect Three Weeks after Administration of Netrin-1

The behavioral assessment of mechanical sensitivity indicated that animals treated three weeks earlier with netrin-1 had significantly higher (i.e., hypersensitive) limb withdrawal rates to mechanical stimulation than vehicle-treated controls (*t*_11_ = 6.1, *p* < 0.0001; Figure 4C).

Electrophysiological in vivo recordings of pronociceptive RVM ON-like neurons (n_Netrin_ = 17, n_Vehicle_ = 32) in lightly anesthetized animals (n_Netrin_ = 5, n_Vehicle_ = 5) indicated that the ongoing discharge rate was higher in animals treated three weeks earlier with netrin-1 (2.0 µg) than in those treated with vehicle (*t*_47_ = 2.1, *p* = 0.04; Figure 4D). Ongoing discharge rates of antinociceptive RVM OFF-like neurons (n_Netrin_ = 19, n_Vehicle_ = 26) in animals (n_Netrin_ = 4, n_Vehicle_ = 6) treated three weeks earlier with netrin-1 were not different from those in vehicle-treated controls (*t*_43_ = 0.1; Figure 4E).

## 3. Discussion

The present study showed that spinal netrin-1, an endogenous chemoattractant, induces in a dose-related manner a long-lasting mechanical hypersensitivity condition. The hypersensitivity effect is submodality-selective as shown by the finding that netrin-1 did not influence heat nociception. The hypersensitivity effect of netrin-1 in the present study is in line with earlier results reporting that netrin-1 is associated with pain in various experimental and pathophysiological conditions [5,6,7,8,9,13] and that the DCC receptor, a key mediator of attractive signaling by netrin-1 [2], may be mediating pronociceptive effects of netrin-1 [5,6,7,11,13]. The present study extends earlier findings by showing that a single intrathecal dose of netrin-1 does not only induce acute hypersensitivity in rodents [7], but it may induce a prolonged neuropathy-like pain hypersensitivity condition lasting at least up to three weeks. In addition, this study shows that among downstream mechanisms mediating the pronociceptive effect induced by netrin-1 is the spinal TRPA1 receptor, a calcium-permeable non-selective cation channel that is a final common pathway for a large number of chemically diverse pronociceptive agonists generated in various pathophysiological pain conditions (see for review [18]). Among potential underlying factors involved in the mediation of the pronociceptive effect of netrin-1 by TRPA1 are that netrin-1 also has proinflammatory actions [21,22,23,24], which might generate endogenous TRPA1 agonists, and the colocalization of netrin receptor DCC with TRPA1 mRNA in some populations of sensory neurons [25,26]. Interestingly, the TRPA1 antagonist effectively attenuated mechanical hypersensitivity induced by netrin-1 both during the early and late phases. Whereas it has been proposed that the evolution of acute pain to chronic pain may be based on changes in the underlying mechanisms [27], the present results suggest that at least partly the same mechanisms mediate acute and chronic pain hypersensitivity induced by netrin-1.

Although TRPC4/C5 has had pronociceptive actions in previous studies (see for review [19]), TRPC4/C5 receptor antagonists failed to attenuate mechanical hypersensitivity induced by netrin-1, except for the weak antihypersensitivity effect during the early phase by the less selective of the currently used antagonists. However, the most prominent TRPC5-induced pronociceptive effects have been demonstrated in primary afferent nerve fibers [28] rather than the spinal cord. In addition to the peripheral neurons, there is earlier evidence that TRPC4/C5 channels are involved in the control of the primary emotion center amygdala [29,30] which is also a key player in the processing and control of affective aspects of pain [31], and which we did not study in the present experiments. Although our present results do not support the hypothesis that spinal TRPC4/C5 had a key role in mediating the hypersensitivity effect induced by netrin-1, this proposal does not exclude the potential pronociceptive role of spinal TRPC4/C5 in other conditions. In line with this caveat, a recent study reported that morphine-induced analgesic tolerance and hyperalgesia were associated with increased expression of spinal TRPC4/C5 [32]. 

The ongoing discharge rate in pronociceptive ON-like pain control neurons of the RVM was increased in hypersensitive animals treated three weeks earlier with netrin-1. The association of the increased discharge of RVM ON-like neurons with behavioral hypersensitivity induced by netrin-1 is in line with the proposal that RVM ON-neurons, through their spinal projections, contributed to the maintenance of pain hypersensitivity in animals treated with netrin-1 in the present study as well as in a number of other pain hypersensitivity conditions demonstrated earlier [33]. Netrin-1, however, did not increase the discharge rate of RVM ON-like neurons within the first 30 min after its administration, nor did netrin-1 induce a discharge rate change in RVM OFF-like neurons that are presumed to have a role in descending antinociception [33]. It should be noted, however, that behavioral experiments were performed in unanesthetized animals, whereas neurophysiological recordings were performed under light anesthesia which influences responses of RVM neurons [34] and may have attenuated responses of RVM neurons to netrin-1 in the present study. Due to high variability in the discharge rates of RVM ON-like neurons of the present study sample, one still needs to be cautious with the conclusion on their role in the hypersensitivity induced by netrin-1 until the present finding has been replicated in a larger study sample. 

The most common finding in clinical examination of neuropathic pain patients is tactile allodynia which can be assessed using a brush or von Frey hairs [35]. In the present study, the administration of netrin-1 into the spinal cord induced hypersensitivity-mimicking tactile allodynia, as revealed by repeated stimulation with von Frey hairs at various levels of the test stimulus force. We did not apply the traditional threshold measurement methods (increasing stimulus intensity until the animal responds, or the “up and down” method [36]), since the traditional threshold measurements ignore potential sensitivity changes that vary with the stimulus intensity. For example, clinical studies indicate that in some neurologic disorders the threshold to mechanical stimulation is not changed, although the sensory response to mechanical stimulation at a suprathreshold intensity is considerably increased [37]. Similarly, experimental animal studies show that some of the descending pain control pathways (that can be activated by centrally acting drugs) modulate only the steepness of the stimulus–response function in the ascending nociceptive barrage; i.e., the nociceptive threshold is not changed, whereas the response to suprathreshold stimulation can be very much changed [38]. Studies that use traditional threshold determination methods fail to reveal the response changes that occur only at a suprathreshold level. With the currently used method, we have previously been able to demonstrate, e.g., a drug effect that takes place predominantly at a threshold but not suprathreshold level (see Figure 4C in ref. [39]), or conversely, a change that takes place predominantly at a suprathreshold but not threshold level (see Figure 3A in ref. [40]). In the current study, however, the changes in pain-related responses following netrin-1 did not vary with test stimulus intensity, as reported in the results section.

The place preference paradigm was used to assess whether mechanical hypersensitivity induced by netrin-1 is accompanied by ongoing pain or unpleasantness [41,42]. Spinally-administered clonidine at the dose that significantly attenuated mechanical hypersensitivity failed to induce place preference. This finding does not support the hypothesis that spinal administration of netrin-1 induced ongoing pain or unpleasantness. Among alternative explanations, however, are that the dose of clonidine-attenuating mechanical hypersensitivity was not sufficient to attenuate ongoing pain, or that the sensitivity of the assay was not enough to detect attenuation of ongoing pain. Moreover, although a single pairing has proved effective for clonidine-induced place preference in chronic neuropathic pain models (e.g., [42,43]), we cannot exclude the possibility that a treatment protocol longer than the currently used two-day drug pairing might have revealed a significant place preference. The finding that netrin-1 induced hypersensitivity to mechanical stimulation but not heat indicates that the hypersensitivity effect was due to action on sensory rather than motor neurons since the motor component of the behavioral assay was the same when testing the two stimulus modalities. A limitation of the present study is that only male animals were studied, although pain sensitivity may vary with sex [44].

The main novel finding of the present study is that TRPA1 is involved in mechanical hypersensitivity induced by the spinal administration of netrin-1. Additionally, increased impulse discharge in pronociceptive pain control neurons of the medulla may contribute to the prolonged hypersensitivity effect induced by netrin-1, although due to large variability in neuronal discharge rates, this finding still needs further confirmation.

## 4. Materials and Methods

### 4.1. Experimental Animals

The provincial government of Southern Finland (Etelä-Suomen aluehallintovirasto, Hämeenlinna, Finland; permission no. ESAVI/41116/2019) had approved the study protocol and the research was performed according to the guidelines of the European Parliament and the Council Directive of 22 September 2010 (010/63/EU), International Association for the Study of Pain [45], and the ARRIVE guidelines [46,47]. Male Han–Wistar rats (Envigo Laboratories, Horst, the Netherlands) weighing 180–230 g at the beginning of the experiments were used. Animals were housed in standard light- and temperature-controlled rooms (lights on 0600–1800 h, temperature 22 ± 2 °C) in individually ventilated plastic cages with free access to tap water and standard laboratory chow. The home cages were environmentally enriched. The animals received commercial pelleted rat feed (CRM-P pellets, Special Diets Services, Witham, Essex, UK) and tap water ad libitum. The experiments were performed in accordance with 3R (replacement, reduction, and refinement) principles, and all efforts were made to limit distress to the animals. Drug treatment conditions were randomized and blinded.

### 4.2. Techniques for Spinal Drug Administrations

For the spinal administration of drugs, an intrathecal (Intramedic PE-10, Becton Dickinson and Company, Sparks, MD, USA) was installed into the lumbar level of the animal’s spinal cord under sodium pentobarbital anesthesia (60 mg/kg i.p.) as described in detail elsewhere [48]. After recovery from anesthesia, the correct placement of the catheter was verified by injecting lidocaine (4%, 7–10 μL followed by a 15 μL of saline for flushing) with a 50-μL Hamilton syringe (Hamilton Bonaduz AG, Bonaduz, Switzerland). Only rats that had no motor impairment prior to the lidocaine injection but had a bilateral paralysis of hind limbs following it were included in the study. The volume of injections was 5 μL followed by a flush with 15 μL of saline. The installation of the catheter was performed one week before the start of the actual testing sessions and the lidocaine test was performed at least three days prior to the start of the drug testing sessions. In the present sample of animals, the success rate with the installation of the catheter was 100%.

### 4.3. Behavioral Testing of Mechanical Hypersensitivity and Heat Nociception

Prior to any behavioral testing, the rats were habituated to the experimental conditions by allowing them to spend 1–2 h daily in the laboratory during 2–3 days. For assessment of tactile allodynia-like hypersensitivity, the hind limb withdrawal threshold evoked by stimulation of the hind paw with monofilaments (von Frey hairs) was determined while the rat was standing on a metal grid. At each time point, the hind paw was stimulated five times with an ascending series of calibrated monofilaments (in netrin group animals 1–26 g, and in healthy controls 1–60 g; North Coast Medical, Inc., Morgan Hill, CA, USA). At each stimulus force, the withdrawal response frequency was determined. An increase in the withdrawal response rate was considered to represent the mechanical hypersensitivity effect. When compared with the traditional determination of the withdrawal threshold value, the currently used method has the advantage that it allows for separately assessing drug effects on withdrawal responses evoked by stimulus forces of threshold and suprathreshold levels. After determining that the hypersensitivity induced by netrin-1 did not vary with the intensity of the test stimulation (Figure 1C and the corresponding statistics in the text of the “Results” section), we reduced redundancy in reporting the results by only showing in most of the graphs the response to the stimulation at the force of 6 g. The reason for choosing the 6 g force was that an earlier study using high-speed videography, statistical modeling, and machine learning showed that in order to evoke withdrawal responses representing pain-like behavior, the test stimulus intensity needs to be at least 4 g in mice [49]. Moreover, the force of 6 g evoked only a weak response in our control animals (Figure 1C) and thereby, it allowed an optimal visualization of increases in response rates. For comparison, we also determined the traditional threshold in grams from psychometric function curves as the stimulus intensity evoking a 50% response rate (see for psychometric function curves e.g., Figure 3 in ref. [50]). 

For the assessment of thermal nociception in the plantar skin of the hind paw, the latency of the heat-induced limb withdrawal response was determined using a radiant heat device (Plantar test model 7370, Ugo Basile, Varese, Italy). To avoid tissue damage, two consecutive measurements at each time point were made at one min intervals. The mean latency at each time point was used in further calculations. The stimulus intensity was adjusted so that the mean baseline latency was 5–7 s and the cut-off latency was 15 s. 

### 4.4. Assessment of Ongoing Pain-Like Behavior in a Conditioned Place Preference Test

Hypersensitivity to test stimulation needs not be associated with ongoing pain [51]. A conditioned place preference test [41,42,43] was used for assessing ongoing pain or unpleasantness and its attenuation by intrathecal administration of clonidine (an α_2_-adrenoceptor agonist; 10 µg). Briefly, rats underwent a three-day habituation, in which they were placed in automated CPP boxes (Place Preference System, San Diego Instruments, Inc., San Diego, CA, USA) with access to all three chambers for 30 min per day during the first two days. The device records time spent in each chamber using a computer-controlled 4 × 16 array of photo beams. Among the differences between the test chambers were the roughness of the floor (rough versus smooth) and the painting of the walls (black triangles versus bars on a white surface). Time spent in each of the boxes was recorded for 15 min on day 2 (D2). Rats that spent more than 720 s in one of the conditioning chambers were eliminated from the study. On the following days (D3 and D4), all rats received a morning injection of saline and were placed in one of the pairing chambers for 30 min. Four hours later, all rats received clonidine (10 μg) and were placed in the opposite chamber for 30 min. On D3, a monofilament test was performed 30 min after saline and 30 min after clonidine treatment to assess whether clonidine influenced responses to mechanical test stimulation. On the fifth day (D5), 20 h following the last drug pairing, animals were placed drug-free in the place preference boxes with access to all chambers. The amount of time spent in each of the two chambers (saline- and clonidine-paired) was automatically registered and used to quantify the conditioning effect of drug treatment. It was expected that if the animal had ongoing pain or unpleasantness that was reduced by clonidine treatment, the animal preferred the clonidine-paired chamber.

### 4.5. Assessment of Anxiety-Like Behavior in the Elevated plus Maze Test

There is abundant evidence indicating that anxiety is among the comorbidities of prolonged ongoing pain and that anxiety-like behavior can also be observed in experimental animal models of chronic pain [52]. To assess whether netrin-1 treatment induces ongoing pain that results in accompanying anxiety, we tested anxiety-like behavior in animals treated with netrin-1 using the elevated plus maze (EPM) test. An EPM consisted of two open arms 45 cm × 10 cm and two closed arms 45 cm × 10 cm × 35 cm with an open roof, elevated to 50 cm from the floor and arranged so that the open arms were opposite to each other [53]. The testing was conducted in a bright environment at a light level of 300 lux. For testing, each animal was placed in the center of the maze and recorded for 5 min by a digital video camera to assess the time spent in each arm. It was expected that if animals have increased anxiety, they would spend less time in the open arm of the device.

### 4.6. Recording of Neuronal Activity in the Rostral Ventromedial Medulla (RVM) 

Electrophysiological microelectrode recordings of RVM cells were performed under anesthesia that was induced by administering 50–60 mg/kg of sodium pentobarbital i.p. Following the induction of anesthesia, the animal was placed in a standard stereotaxic frame according to the atlas of Paxinos and Watson [54]. Anesthesia was continued by i.p. injections of pentobarbital at intervals of 30 min and at the dose of 15–20 mg/kg. The level of anesthesia was frequently monitored by assessing the size of the pupils, general muscle tone, and reflex responses to noxious pinching. Supplemental doses of sodium pentobarbital were administered as required. The rats breathed spontaneously and the body temperature was maintained within a physiological range with a warming blanket. Peripheral perfusion was checked by examining the color of the ears and extremities.

The skull was exposed and a hole was drilled for the placement of a recording electrode in the RVM (anteroposterior [AP]: 2.0–2.8 mm from the interaural line; mediolateral [ML]: 0–1 mm; dorsoventral [DV]: 8.9–11 mm from the dura mater [54]). Neuronal activity in the RVM was recorded extracellularly with lacquer-coated tungsten electrodes (impedance 5–7 MΩ at 1 kHz; FHC Inc., Bowdoin, ME, USA). The signal was amplified and filtered using standard techniques. Data sampling was performed with a computer connected to a CED Micro 1401 interface and using Spike2 software (Cambridge Electronic Design, Cambridge, UK). Spike2 software classifies waveform shapes based on full wave templating, and in the offline analysis, the template matching can be complemented by clustering using principal component analysis, which allows separately evaluating multiple identified units in a single recording session.

**Figure 5:**
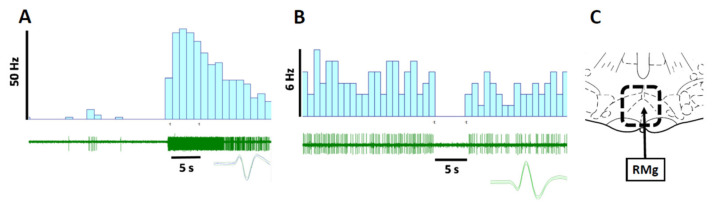
Examples of original recordings of an ON-like neuron (**A**) and an OFF-like neuron (**B**), and the recording area in the rostral ventromedial medulla (**C**). In (**A**,**B**), the uppermost graph shows a peristimulus time histogram, the middle one an original recording, and the lowermost a graph template of the studied neuron. The horizontal time bar indicates the time and duration of the noxious tail pinch used to classify the neuron based on its stimulus-evoked response. In (**C**), the area surrounded by the dotted line in the section that is 2.3 mm caudal to the ear bar indicates the area of the verified recording sites. RMg = raphe magnus.

During recordings, the microelectrode was first lowered according to the stereotaxic coordinates into the RVM. When neuronal firing was observed, spontaneous ongoing activity was first assessed. Before starting the actual recording of cells, the deep level of anesthesia needed for the surgical procedures was allowed to lighten to a level where the animal did not have any spontaneous limb movements but noxious stimulation caused a brief flexion reflex with no other behavioral responses. The RVM cells were classified based on the concurrently-assessed RVM cell and movement response to a noxious pinch of the tail using a classification scheme modified from the original one [33]; due to the modification of the original classification scheme, the terms ON-like and OFF-like are used in this study, instead of ON- and OFF-cells. RVM cells that gave an excitatory discharge > 20% to the tail pinch were classified as pronociceptive ON-like cells (Figure 5A). RVM cells, in which the ongoing discharge rate was inhibited by >20% to the tail pinch, were classified as antinociceptive OFF-like cells (Figure 5B). RVM cells that did not respond to noxious heat were considered NEUTRAL cells. NEUTRAL cells were not included in this study. After the characterization of the RVM cell as a presumably pronociceptive RVM ON-like or a presumably antinociceptive RVM OFF-like cell, its ongoing discharge rate was determined in the studied conditions. In the same recording session, multiple neurons could be recorded and analyzed separately. At the end of the recording, the recording site was marked electrolytically; the animal was given a lethal dose of sodium pentobarbital (200 mg/kg), the brain removed, and the recording sites verified in coronal brain sections.

### 4.7. Drugs

Netrin-1 (recombinant mouse netrin # 1109-N1-025/CF) was purchased from Bio-Techne Ltd. (Abingdon, UK), dissolved in saline, and administered at a dose range (0.2–2.0 µg/5 µL) that has proved pronociceptive following spinal administration in mice [7]. Chembridge-5861528 (Chem; a derivative of HC-030031), which was synthesized by ChemBridge Corporation (San Diego, CA, USA) was used as a TRPA1 channel antagonist at a dose of 10 µg that has had a significant antihypersensitivity effect in various pain models following intrathecal administration [39]. Two different TRPC4/C5 antagonists used in the study were ML-204 and HC-070. ML-204 [55] was purchased from Sigma-Aldrich (St. Louis, MO, USA). ML-204 was dissolved in dimethylsulfoxide (DMSO, 100%) and administered at the dose of 10 µg that has had a significant antihypersensitivity effect when administered into the central amygdaloid nucleus [56]. HC-070 [57] was synthesized by Orion Pharma (Espoo, Finland) and it was dissolved in 10% DMSO + 5% CremophorEL + 85% saline. The HC-070 dose of 19 ng was chosen based on a preliminary study showing that at this dose, spinally administered HC-070 alone had no effect on monofilament-induced withdrawal response in control animals (response to 6 g from 16 ± 8% to 17 ± 8%, mean + SD, n = 6; *t*_5_ = 1.0, *p* = 0.36), nor did the vehicle of HC alone induce a change (response to 6 g from 13 ± 4% to 16 + 6%, n = 6; *t*_5_ = 1.0, *p* = 0.36), whereas at a dose of 190 ng HC-070 alone significantly increased the response rate (response to 6 g from 16 + 8% to 40 ± 12%, n = 6; *t*_5_ = 3.8, *p* < 0.013). Pregabalin (the established control analgesic) and clonidine hydrochloride (α_2_-adrenoceptor agonist) were purchased from Sigma-Aldrich and used at doses that have had a significant antinociceptive effect after spinal administration in earlier studies (pregabalin 30 µg [58]; clonidine 10 µg [42]).

### 4.8. Course of the Study

**Figure 6:**
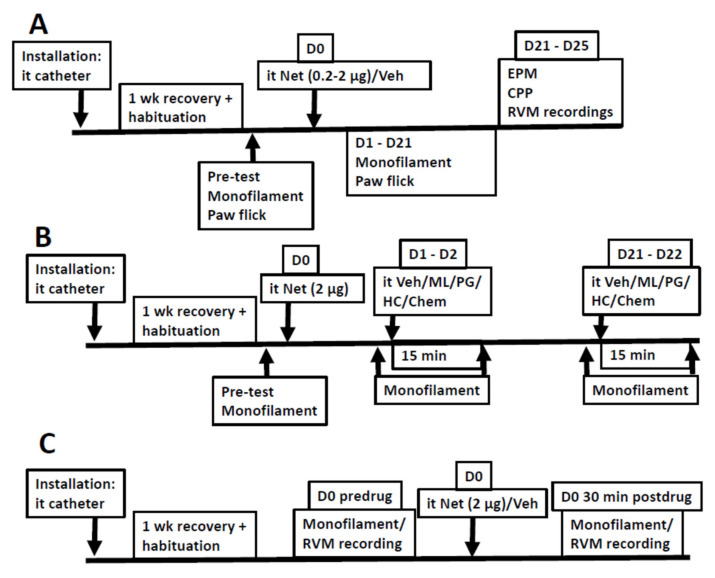
Schematic time course of the experimental procedures when assessing basic characteristics of the prolonged hypersensitivity (**A**), reversal of hypersensitivity (**B**), and acute hypersensitivity (**C**). Chem = Chembridge-5861528; CPP = conditioned place preference; D0–D21 = Day of testing; EPM = elevated plus maze; HC = HC-070; it = intrathecal; ML = ML-204; Net = Netrin-1; PG = Pregabalin; RVM = rostral ventromedial medulla; Veh = Vehicle.

Figure 6 describes the course of the study. All experiments started with the installation of a chronic intrathecal catheter one week before the actual experiments. In all experiments, the animals were habituated to the experimental conditions during the one-week recovery period after the catheter installation. When studying basic characteristics of the hypersensitivity, vehicle or netrin-1 was administered at a dose of 0.2 or 2.0 µg on D0 (Figure 6A). Mechanical sensitivity (monofilament test) and heat nociception (paw flick test) was determined before drug administration and daily for the first three days, after which testing was performed once a week. Anxiety-like behavior (EPM test) and ongoing pain-like behavior (CPP test) were assessed. Moreover, recordings of RVM ON-like and OFF-like neurons were performed in the third week (Figure 6A). 

Attempts to attenuate hypersensitivity induced by 2.0 µg of netrin-1 were performed in the early phase (one to two days after administration of netrin-1), or the late phase (days 21–22) (Figure 6B). During both the early and late phases, mechanical sensitivity was assessed before and 15 min after administration of the studied compound (vehicle/ML-204/pregabalin/HC-070/Chembridge-5861528).

The acute effect induced by administration of netrin-1 at the dose of 2.0 µg was assessed on mechanical sensitivity with behavioral methods in one group of awake animals and on the discharge rate of RVM ON-like neurons in a separate group of lightly anesthetized animals (Figure 6C). In the behavioral experiment, mechanical sensitivity was assessed before and 30 min after the administration of netrin-1. In the electrophysiological experiment, after the induction of anesthesia, the placement of the recording electrode, and finding an ON-like neuron in the RVM, the ongoing discharge of the neuron was assessed before and 15 min after administration of netrin-1. In the acute recording condition, unlike in the chronic hypersensitivity phase recording condition, OFF-like neurons were not studied. This is because in the acute recording condition it was possible to study only one recording site/condition in each animal; i.e., recording of the same single neuron (or more, if more than one unit could be separately isolated in the same session) before and after administration of netrin-1. To reduce the number of animals, the focus in the acute recordings was on ON-like neurons, since RVM ON-cells have been shown to play a role in various acute hypersensitivity conditions [33]. In contrast, in the chronic phase, it was possible to study multiple recording sites and multiple neurons in each animal, allowing assessment of the discharge of both ON-like and OFF-like neurons. 

### 4.9. Statistics

A one-way or two-way mixed-design analysis of variance followed by a Bonferroni-corrected *t*-test was used when comparing three or more groups. Student’s *t*-test (paired one, when appropriate) was used when comparing two groups; *p* < 0.05 was considered to represent a significant difference. 

## 5. Conclusions

The present results support the hypothesis that netrin-1 in the spinal cord induces a selective mechanical hypersensitivity effect that may, at least partly, be maintained by downstream activation of spinal TRPA1. Moreover, the increased discharge rate of pronociceptive medullary pain control neurons may also contribute to hypersensitivity induced by netrin-1, although this finding still needs further corroboration in a larger sample of neurons.

## Data Availability

Not applicable.
